# Nighttime smartphone use and changes in mental health and wellbeing among young adults: a longitudinal study based on high-resolution tracking data

**DOI:** 10.1038/s41598-022-10116-z

**Published:** 2022-05-15

**Authors:** Agnete Skovlund Dissing, Thea Otte Andersen, Andreas Kryger Jensen, Rikke Lund, Naja Hulvej Rod

**Affiliations:** 1 Section of Epidemiology, Department of Public Health, University of Copenhagen, Oester Farimagsgade 5, Postbox 2099, 1014 Copenhagen, Denmark; 2 Section of Biostatistics, Department of Public Health, University of Copenhagen, Oester Farimagsgade 5, Postbox 2099, 1014 Copenhagen, Denmark; 3 Section of Social Medicine, Department of Public Health, University of Copenhagen, Oester Farimagsgade 5, Postbox 2099, 1014 Copenhagen, Denmark; 4 Center for Healthy Ageing, Faculty of Health Sciences, University of Copenhagen, Blegdamsvej 3B, 2200 Copenhagen, Denmark

**Keywords:** Epidemiology, Psychiatric disorders, Risk factors

## Abstract

Frequent nighttime smartphone use can disturb healthy sleep patterns and may adversely affect mental health and wellbeing. This study aims at investigating whether nighttime smartphone use increases the risk of poor mental health, i.e. loneliness, depressive symptoms, perceived stress, and low life satisfaction among young adults. High-dimensional tracking data from the Copenhagen Network Study was used to objectively measure nighttime smartphone activity. We recorded more than 250,000 smartphone activities during self-reported sleep periods among 815 young adults (university students, mean age: 21.6 years, males: 77%) over 16 weekdays period. Mental health was measured at baseline using validated measures, and again at follow-up four months later. Associations between nighttime smartphone use and mental health were evaluated at baseline and at follow-up using multiple linear regression adjusting for potential confounding. Nighttime smartphone use was associated with a slightly higher level of perceived stress and depressive symptoms at baseline. For example, participants having 1–3 nights with smartphone use (out of 16 observed nights) had on average a 0.25 higher score (95%CI:0.08;0.41) on the Perceived stress scale ranging from 0 to 10. These differences were small and could not be replicated at follow-up. Contrary to the prevailing hypothesis, nighttime smartphone use is not strongly related to poor mental health, potentially because smartphone use is also a social phenomenon with associated benefits for mental health.

## Introduction

Mental health problems are recognized as a major burden of disease, and a substantial number of adults struggle with mental health problems^1^. In many Western societies, an increase in poor mental health has been detected in particular among young adults during the last decades^2,3^. During the same period advances in smartphone technologies have become increasingly available causing widespread and round-the-clock use of smartphones especially among the younger generations^4,5^. The parallel increase in both smartphone use and poor mental health in western societies is striking and draws attention to excessive smartphone use as a potential risk factor for poor mental health^6,7^. In particular, the frequent nighttime smartphone use may disturb sleep and thereby adversely affect mental health^8^.

As part of the sustainable development goals, the United Nations has proclaimed to promote mental health and wellbeing by 2030^9^. However, research is yet to identify modifiable risk factors that can be easily targeted in mental health interventions. Considering the widespread nighttime smartphones use even small effects on mental health can have major public health implications. Specifically preventing nighttime smartphone use may be a tangible population behaviour change as it requires a relatively simple change for the individual such as turning off the smartphone at night or leaving the smartphone out of the bedroom^10^. Hence, it is relevant to investigate whether and how nighttime smartphone use may affect mental health.

The use of light-emitting electronic devices before and during the sleep period is likely to stimulate cognitive arousal and delay the release of the sleep hormone melatonin affecting both sleep onset latency and sleep perceptions^11–14^. Prolonged sleep disruptions are likely to interfere with mental restitution^15,16^ and mood^17^, and several studies have shown that poor sleep plays an important etiological role in the development of poor mental health working through changed emotional regulation and neuro-biological interaction^18–20^. Poor sleep quality has shown to either affect or exacerbate feelings of perceived stress^21^, which may over a longer period develop into depressive symptoms^22^. Further, sleep deprivation may also hamper daily functioning and the ability and energy to engage in meaningful activities, which are likely to affect overall life satisfaction and feelings of being socially connected. While it is less likely that sleep disruption from technology use plays a key role in the aetiology of severe mental disorders requiring prolonged clinical intervention, we hypothesise that the general mental wellbeing such as stress perceptions, life satisfaction, depressive symptoms, and feelings of social isolation may be affected.

In a prior study using data from a population-based citizen science sample of 24,856 Danish adults, we showed that 81 percent of the men and 88 percent of the women aged 16–25 years reported to use their smartphone before falling asleep a few times a week or more, and one-third reported to use their smartphone during the sleep period^5^. We also showed that phone use during the sleep period relative to other smartphone behaviours had the strongest association with poor sleep, and hence this behaviour might pose a risk factor for poor mental health.

A systematic review shows that nighttime phone use is related to measures of poor sleep^8^. However, most studies in the area are conducted among children and adolescents, and only a few studies have investigated nighttime smartphone use in relation to mental health in adult populations^6,23^. We have only been able to identify one study that reported on the longitudinal relationship between nighttime smartphone use and mental health in an adult population, and this study found no associations at follow-up^6^. Further, studies in the field are predominately based on self-reported measures of both nighttime smartphone use and mental health in cross-sectional studies limiting the validity of these findings. Hence, more research is needed to investigate the relationship between nighttime smartphone use and mental health in adult populations—in particular among young adults where nighttime smartphone use is very frequent^5,24^. In order to overcome measurement bias due to self-reports, phone tracking has been proposed as an alternative strategy, but this approach has mostly been applied in small samples (N ~ 100) and with a short observation period^25,26^. Although this measurement method is difficult to apply in large population-based studies, it has the advantage of being recorded independently from outcome(s) of interest with a very high information resolution.

The aim of this paper is to investigate whether nighttime smartphone use is associated with the risk of poor mental health and wellbeing, i.e. loneliness, depressive symptoms, perceived stress, and low life satisfaction. We will use a unique dataset that allows us to study multiple behavioural dimensions of nighttime smartphone use in relation to mental health using longitudinal high-dimensional tracking data from smartphones in a sample of 815 young adults.

## Material and methods

### The Copenhagen Network Study

In 2013, 3329 undergraduate students at the Danish Technical University were invited to participate in the Copenhagen Network Study^27^. In total, 979 students (29%) accepted the invitation (60% were freshmen students). All participants signed an informed consent. The students participating in the study were given a smartphone (LG Nexus 4) which was running customised software continuously recording information on all call and text message interactions (not content). The students were required to insert their private SIM-card into the provided smartphone to make it their primary phone and to respond to a baseline questionnaire. The questionnaire was presented using a custom-built web application. Facebook data (Facebook friends, likes, and status updates) were captured by asking the participants to authorise data collection from their Facebook account using access tokens. All data were linked at the individual level, and we used data collected in a four-week period starting one week after the participants first activated their smartphone.

**Figure 1:**
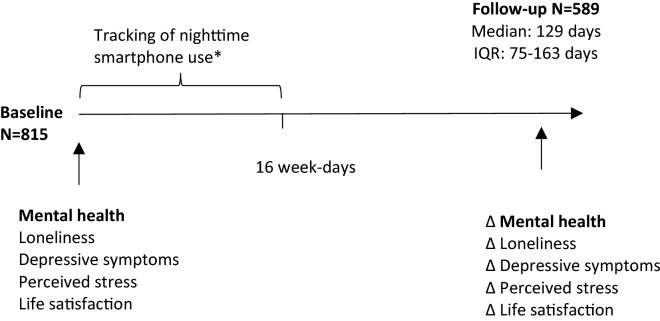
Overview of study design and data collection in the Copenhagen Network Study. *Data from the first week of smartphone use were excluded.

We excluded the first week of phone use to allow for adjustment to the new phone. As we assumed the sleep patterns over the weekends to be considerably different from weekdays, we only used data from weekdays (Monday through Thursday) from the four-week period. We excluded individuals with no information on mental health (N = 59) and with missing phone recordings (N = 105) yielding a total sample of 815 individuals who were included in the analyses at baseline. After approximately four months (interquartile range (IQR): 75–163 days), 589 participants (72% of baseline population) responded to a follow-up questionnaire and of these between 47–51 participants had missing values in the four outcomes of interests. More men (30%) than women (22%) were lost to follow-up, but loss to follow-up was unrelated to nighttime smartphone use, mental health, and age^28^. See Fig. 1 for an overview of the study design.

The experiments done in this study are in accordance with the relevant guidelines and regulations.

(Approval from the Danish Data Protection Agency, number: 2012–41-0664). The current study does not require approval by the National Committee on Health Research Ethics by Danish law.

### Tracked nighttime smartphone use: number of nights with less than six hours of consecutive sleep

We recorded the exact timing of smartphone activities from one hour before self-reported usual weekday bedtime throughout the self-reported sleep period calculated from self-reported usual weekday bedtimes and rise times. We recorded each of the following smartphone activities during the self-reported sleep period which all required active engagement and thus indicating that the participant was awake: received ingoing calls, outgoing calls, outgoing text messages, uploaded Facebook status-reports and ‘liking’ a post on Facebook. Building on this information including more than 250,000 records of nightly smartphone activity, we determined for each night the longest consecutive passive period without smartphone activity within the self-reported sleep period. As it is well established in the literature that less than six hours of sleep is related to higher risk of morbidity and mortality^29^, we derived a variable counting the number of nights out of the 16 week days where participants had less than six consecutive hours due to smartphone activity during the self-reported sleep period. The variable was grouped into categories indicating the number of nights with less than six consecutive hours of no smartphone activity: 0 Nights, 1–3 Nights, > 3 Nights.

### Mental health and wellbeing outcomes

*Loneliness* was evaluated with a Danish version of the UCLA loneliness scale; a 20-item inventory measuring individual’s subjective feelings of loneliness and social isolation. A score between 0 (least lonely) to 60 (most lonely) was obtained^30^. *Depressive symptoms* were measured using the Major Depression Inventory (MDI)^31^, which is a self-reported 12-item mood questionnaire evaluating depressive symptoms on a 5 point Likert scale. The total depressive symptoms severity scale ranges from 0 to 50, where a score of 50 indicates the most severe level of depressive symptoms. *Perceived stress* was measured using a Danish consensus translation of the Perceived Stress Scale (PSS)^32^. The 10-item PSS instrument was designed to measure the degree to which everyday situations are appraised as being stressful measured using a score ranging from 0 to 40, where a score of 40 indicates the highest level of perceived stress. The Danish consensus translation of the PSS has shown good reliability, internal consistency (ICC = 0.87, Cronbach’s alfa = 0.84) and validity^33^. *Life Satisfaction* was evaluated with the Satisfaction with Life Scale (SWLS): This 5-item inventory measures global satisfaction with one's life. Scores range from 5 (lowest life satisfaction) to 25 (highest life satisfaction)^34^. To increase ease of interpretation and comparability between outcomes, all measures were re-scaled from their original scale to a scale going from 0 to ten.

### Co-variates

*Gender, age* and *cohabitation* (Do you live alone; yes, no) were self-reported in the baseline questionnaire. The personality traits *neuroticism and extroversion* were measured at baseline with the 44-item version of the Big Five Inventory (BFI)^35^. These traits are strongly related to both smartphone use and mental health^35,36^. *The social network score* was measured at baseline with the following item from the Copenhagen Social Relations Questionnaire^37^ indicating a social contact frequency with six different social roles: How often are you together with any of the following people who you do not live with? Mother, father, siblings, extended family, partner, and friends (Response code: Several days a week; About once a week; One to three times a month; Less often than once a month; Never; Have no; Live with). Participants reporting “live with” were grouped in the highest contact frequency category, and participants reporting “Have no” were grouped in the lowest contact frequency group. The contact frequency from the six roles was summed to indicate a measure of total contact frequency and hence reflect both diversity and frequency in social interactions. The social network is strongly related to mental health^38^ and smartphone use as smartphone use may reflect interaction with an underlying social network^39^.

### Analytical strategy

First, we explored characteristics of the study population. Second, we conducted two separate cross-sectional multiple linear regression analyses for the associations between the nighttime smartphone use and the four mental health outcomes at baseline in the full population (N = 815) and at follow-up (N_UCLA_ = 542, N_MDI_ = 538_,_ N_PSS_ = 540_,_ N_SWLS_ = 541). The analyses were adjusted for age, gender, cohabitation status, social network score, and the personality traits neuroticism and extroversion which were identified as potential confounders based on the framework of directed acyclic graphs^40^. Third, we assessed the associations between nighttime smartphone use and changes in the four mental health outcomes from baseline to follow-up approximately four months later by including the baseline mental health outcomes in addition to the identified confounders in a follow-up model. F-tests and associated *p*-values were calculated for the final models evaluating the significance of nighttime smartphone use and the considered mental health outcome.

In a sensitivity analysis, we restricted the baseline models to the population at follow-up to evaluate whether the observed changes were due to effects of underlying different populations. In addition, we included an interaction term between the variable of nighttime smartphone use and follow-up time (grouped in three time-bands: less than 75 days, between 750 and 150 days, and above 150 days) in order to assess whether the effect of nighttime smartphone use differed between participants with short and long follow-up time. All analyses were conducted in the statistical software R.

### Ethical approval

Data used in the manuscript are from the Copenhagen Networks study which has been approved by the Danish Data Protection Agency (DDPA) Journal nr 2012–41- 0664. DDPA is the relevant legal entity in Denmark. Informed consent was obtained from all individual participants included in the study.

## Results

### Characteristics of the study population

Table 1 shows the summary statistics for the study population at baseline. The mean age was 21.6 years and the majority of the study population was men (77.3%), which roughly corresponded to the gender and age distribution at the Danish Technical University (men: 68%, mean age: 21). Participants who had more than three nights with less than 6 h of consecutive sleep due to smartphone use were more often women and on average scored higher on the extroversion personality dimension compared to participants with a lower number of nights with smartphone use.

**Table 1:** Characteristics of the study population 815 participating young adults (N = 815).

	Nighttime smartphone use			Total	*P*-value
	0 Nights	1–3 Nights	> 3 Nights		
Age, mean (sd)	21.6 (2.8)	21.7 (2.5)	21.2 (1.5)	21.6 (2.6)	0.52
Gender, *n* (%)					
Woman	102 (21.3)	68 (23.3)	15 (33.3)	185 (22.7)	
Man	376 (78.7)	224 (76.7)	30 (66.7)	630 (77.3)	0.18
Co-habitation, *n* (%)					
Yes	294 (61.5)	166 (56.8)	28 (62.2)	488 (59.9)	
No	184 (38.5)	126 (43.2)	17 (37.8)	327 (40.1)	0.42
Social network score *n* (%)					
0–9	60 (12.6)	52 (17.8)	8 (17.8)	120 (14.7)	
10–14	208 (43.5)	129 (44.2)	20 (44.4)	357 (43.8)	
15–19	182 (38.1)	93 (31.8)	13 (28.9)	288 (35.3)	
20–24	28 (5.9)	18 (6.2)	4 (8.9)	50 (6.1)	0.33
Personality, mean (sd)					
Extroversion	3.3 (0.7)	3.4 (0.7)	3.5 (0.7)	3.4 (0.7)	0.039
Neuroticism	2.4 (0.6)	2.4 (0.6)	2.4 (0.7)	2.4 (0.7)	0.75
Mental health, mean (sd)					
Perceived stress^a^	3.1 (1.4)	3.3 (1.4)	3.3 (1.6)	3.2 (1.4)	0.097
Depressive symptoms^b^	5.1 (2.1)	5.4 (2)	5.1 (2.2)	5.2 (2)	0.13
Loneliness^c^	3.0 (1.3)	2.9 (1.3)	2.6 (1.3)	3 (1.3)	0.053
Satisfaction with life^d^	7.1 (1.7)	7 (1.7)	7.4 (1.8)	7.1 (1.7)	0.30

SD = standard deviation. ^a^Rated on a scale of 0–40—shown in rescaled version 0–10, ^b^Rated on a scale of 0–50—shown in rescaled version 0–10, ^c^Rated on a scale of 0–60—shown in rescaled version 0–10, ^d^Rated on a scale of 5–25—shown in rescaled version 0–10.

### Nighttime smartphone use and mental health

**Figure 2:**
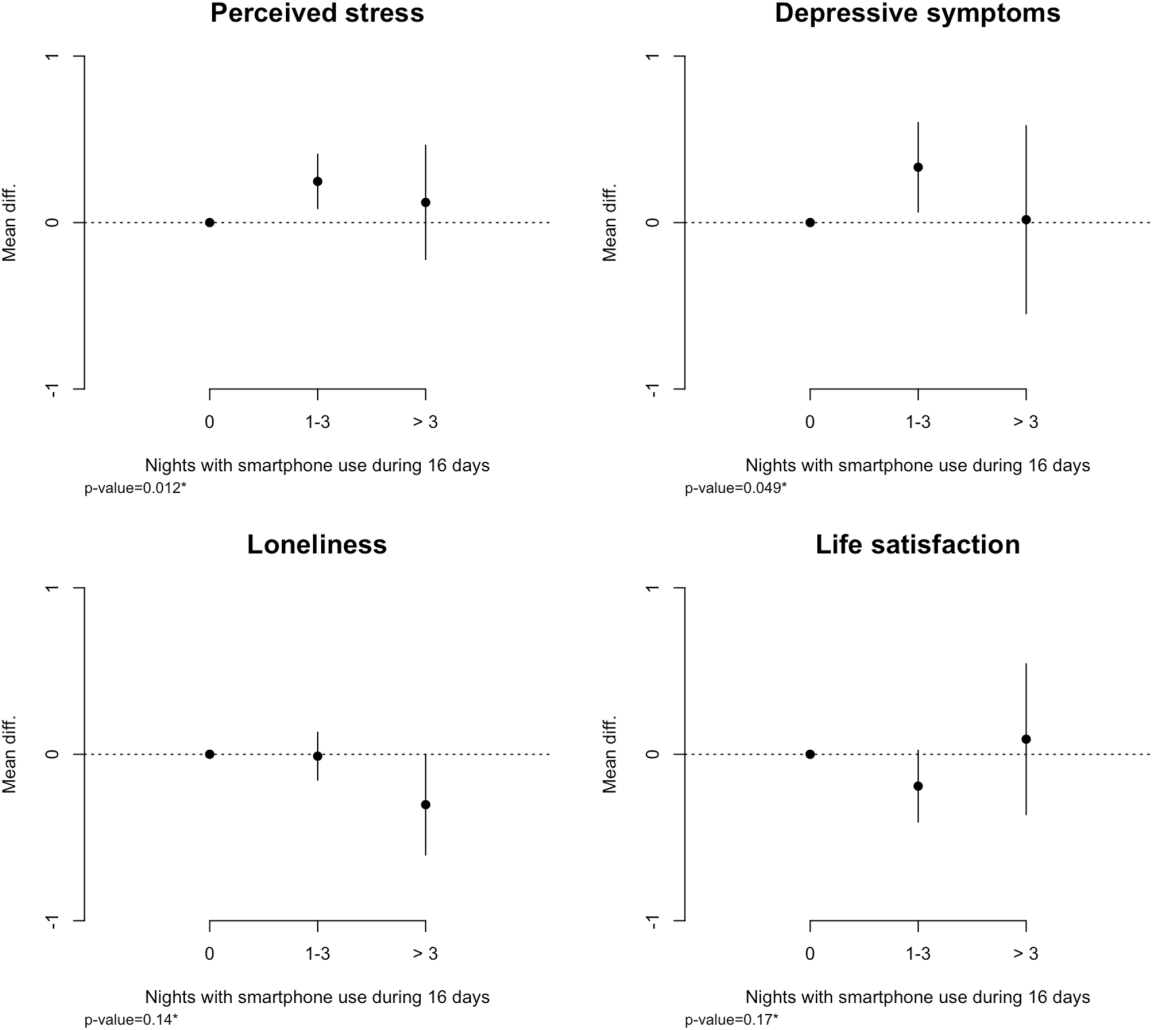
Baseline associations between nighttime smartphone use and mental health. All estimates are adjusted for age, gender, personality (neuroticism and extroversion), co-habitation, social network score. *P-value from F-test. Mean differences denotes the estimated mean difference from the reference group (0 Nights) on a scale ranging from 0 to 10. Error bars corresponds to a 95% confidence interval for the estimated mean difference.

Figure 2 shows the baseline associations between nighttime smartphone use and mental health outcomes. The figure shows that having nights with less than six hours of consecutive sleep is associated with higher levels of perceived stress and depressive symptoms. We found that participants having 1–3 nights with smartphone use had on average a 0.25 points higher score (95%CI:0.08;0.41) on the PSS scale from 0 to 10, and a 0.33 (95%CI: 0.06;0.60) points higher score on the MDI scale ranging from 0 to 10 compared to participants with no smartphone interrupted nights. The results were less clear for loneliness and life satisfaction, but nighttime smartphone use appeared to be associated with a lower level of loneliness—participants having > 3 nights with nighttime smartphone use scored on average 0.30 lower (mean diff. − 0.30 95%CI: − 0.61; − 0.001) on the UCLA loneliness scale ranging from 0 to 10 than participants not using their phone during the sleep period. All mentioned estimates were adjusted for potential confounders.

**Figure 3:**
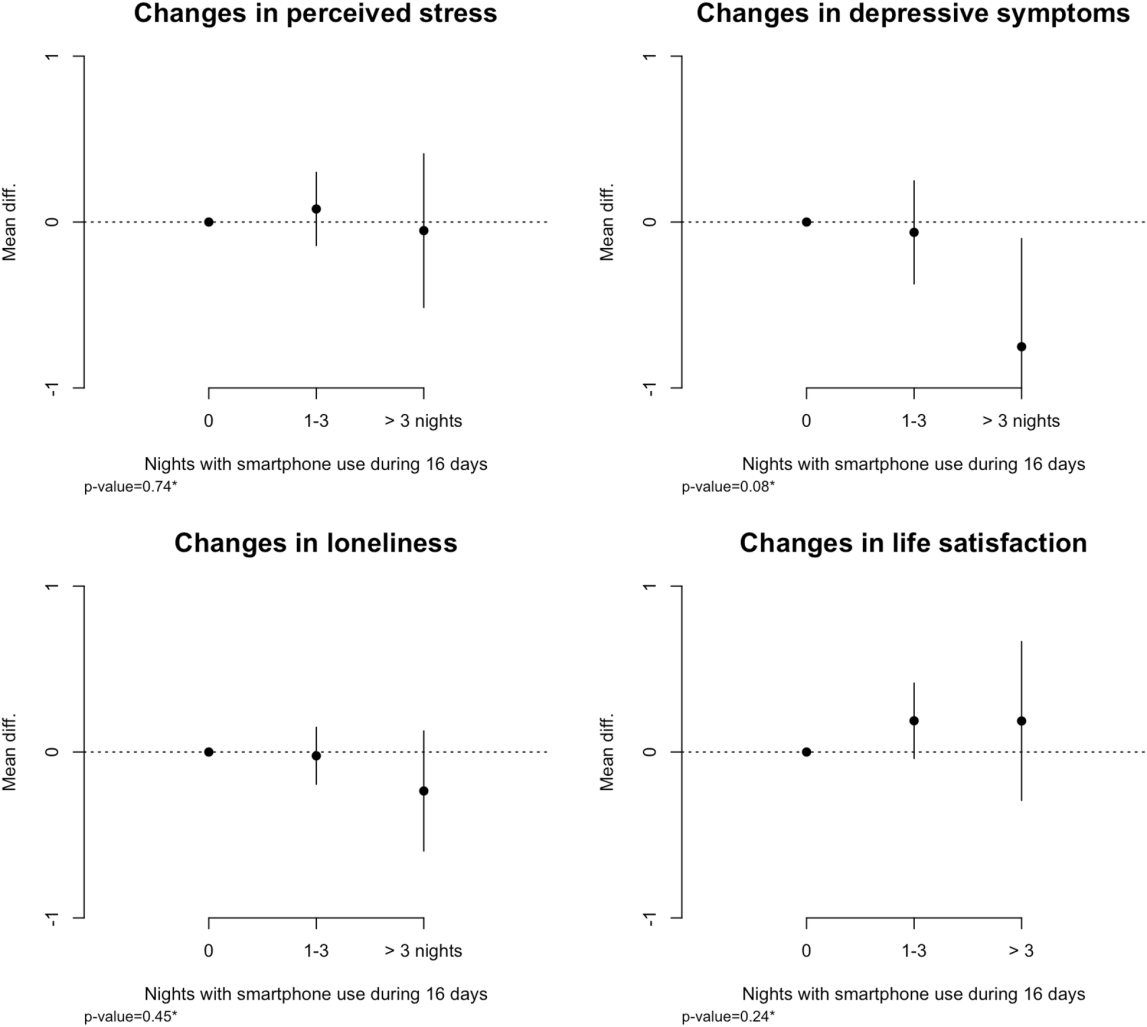
Associations between nighttime smartphone use and changes in mental health at follow-up. All estimates are adjusted for age, gender, personality (neuroticism and extroversion), co-habitation, and social network score. *P-value from F-test. Mean differences denotes the estimated mean difference from the reference group (0 Nights) on a scale ranging from 0 to 10. Error bars corresponds to a 95% confidence interval for the estimated mean difference.

Figure 3 shows the associations between nighttime smartphone use and changes in mental health outcomes at follow-up. Overall, there were no clear associations between smartphone interrupted sleep and changes in perceived stress, loneliness and life satisfaction over an average four-month follow-up period. Contrary to our hypothesis, more than three nights of nighttime smartphone use was associated with a small decrease in depressive symptoms from baseline to follow-up compared with participants who did not use the smartphone at night. The number of participants in this group was small (N = 45), and hence this result should be interpreted with caution. Limiting the baseline associations to participants who responded to the follow-up questionnaire did not change the results considerably, and the observed effects did not differ by length of follow-up. See supplementary information for a full table of all estimates and results from sensitivity analyses.

## Discussion

In a longitudinal study of 815 young adults, we leveraged objective tracking data from more than 250,000 data points, and we found that nighttime smartphone use was associated with slightly higher levels of perceived stress and depressive symptoms at baseline. These differences were small and could not be replicated in longitudinal analyses. Rather, it appeared that frequent nighttime smartphone use was associated with a small decrease in depressive symptoms over time.

An Australian cross-sectional survey of 397 adults showed that sending and receiving calls and texts after lights out and being woken by phone use were associated with lowered mood^23^. Likewise, a Swedish study of 4156 young adults (age: 20–24 years) found associations between self-reported nighttime awakenings by the phone and perceived stress and depressive symptoms. However, the study did not find longitudinal associations between these variables at follow-up one year later^6^. Although conducted among adolescents (1101 adolescents aged 13–16 years), another Australian study is worth mentioning as it is one of few studies considering changes in mental health similar to the current study. They found cross-sectional associations between nighttime smartphone use and depressive symptoms, but when they considered changes in nighttime smartphone use and subsequent changes in depressive symptoms, this relationship was attenuated^41^. Although the present study was carried out using a different measurement method of nighttime smartphone use, we report similar findings. In an earlier cross-sectional study also using objective tracking data, we found that nighttime smartphone use was not associated with depressive symptoms^24^. Combined, these findings do not support the prevailing hypothesis that nighttime smartphone use is a strong risk factor for poor mental health among adults, and they underpin the complexity of teasing out causal inference in the area of nightly smartphone use, sleep and mental health as these factors are highly interlinked.

In the same vein, it is important to keep in mind that the type of activity considered in the current study indicated social interaction. It is well known that social relations and social interactions are generally beneficial to mental health^38^. The current study population consisted of young adults newly enrolled at university where forming social networks and engaging in social interaction are crucial for mental health and wellbeing. Even though nighttime smartphone use is likely to disturb sleep, it is possible that the beneficial effects from having social contact overrides the negative consequences of having disturbed sleep. We tried to accommodate this dual effect by adjusting for participants’ social network score at baseline, but may not fully capture this social element. An American cross-sectional survey conducted among 308 frequent smartphone users suggested that anxiety was related to consumption-based smartphone use (e.g., news consumption, entertainment, relaxation) rather than social smartphone use^42^. This highlights the importance of considering the type and content of smartphone activity as different activities with potentially different implications for mental health. We suggest future studies to consider a broader range of nighttime smartphone activities (screen use, passive usage, active usage) in order to investigate the mental health effects of smartphone activities.

### Strengths and weaknesses

We were able to objectively track the nighttime smartphone use of more than 800 young adults and relate this behaviour to changes in several validated mental health outcomes. This is a particular advantage as most previous studies using tracking data in combination with survey data have only included relatively small samples in a cross-sectional set-up^25,26^.

We aimed at only considering smartphone activities that indicated that the participant was awake during their self-reported sleep period, e.g. only *received* incoming calls and not just incoming calls. Still, it is difficult to know whether the participants had sleep interruptions due to the incoming call or whether they were already awake because of existing sleep problems that may have affected the mental health status prior to the study. In an earlier study conducted in the same study population (restricted to first-year students), we found that mental health was important for daily smartphone communication and social interaction^43^. In the current study, we tried to accommodate the potential reversed causation by considering changes in mental health occurring after baseline. The small differences in mental health detected at baseline could suggest that reverse causality mechanisms may be a valid explanation. Further, it should be mentioned that it was not possible to consider all relevant activities carried out using the phone, and the recorded nightly smartphone activity is likely to be underestimated. We prioritized recording activities from the most commonly used social media platform among young adults in Denmark during the study period 2013–14^44^. In relation to this, it should be noted that the exposure group having most nights with smartphone use (> 3 nights) only consisted of 45 participants and hence, results from this group should be interpreted with caution. Further, it should be noted that we did not have information on the participants’ bedtimes during weekends and we could therefore only consider sleep disruptions during weekdays. The observed effects could potentially differ between men and women. Due to the low proportion of women in this population, we refrained from investigating stratified gender effects, but we suggest future studies to explore this aspect further.

### Conclusions

Contrary to the prevailing hypothesis, nighttime smartphone use was not strongly associated with poor mental health, possibly because smartphone use is also a social phenomenon with beneficial effects on mental health. Further research is warranted in order to confirm these findings preferably designs distinguishing between nightly social and consumption-related smartphone use.

## Supplementary Information

Supplementary Information.

## Data Availability

The full data set contains personally identifiable telecommunication patterns and survey data. According to the Act on Processing of Personal Data, such data cannot be made available in the public domain. The authors confirm that the data is available upon request to all interested researchers under conditions stipulated by the DDPA. Data inquiries should be addressed to the Social Fabric steering committee (http:// sodas.ku.dk), to be reached at ddl@econ.ku.dk.
