# Supplementary material for: Nighttime smartphone use and changes in mental health and wellbeing among young adults: a longitudinal study based on high-resolution tracking data
Source: Sci Rep. 2022 May 15;12:8013. doi: 10.1038/s41598-022-10116-z (PMC9108093; doi:10.1038/s41598-022-10116-z)
Supplement: Supplementary file 1 — Supplementary Information. [file 41598_2022_10116_MOESM1_ESM.docx]

**Supplementary information**

**Associations between nighttime smartphone use and mental health and wellbeing at baseline and follow-up. Table S2 includes results from interaction analysis between smartphone use and follow-up time.**

| **Table S1: Associations between nighttime smartphone use and mental health and well-being at baseline** | | | | | | | | | |  |  |  |  |  |  |  |
| --- | --- | --- | --- | --- | --- | --- | --- | --- | --- | --- | --- | --- | --- | --- | --- | --- |
|  | **Perceived stress** | | |  | **Depressive symptoms** | | |  | **Loneliness** | | |  | **Satisfaction with life** | | |  |
| Nights with smartphone use | *coef.* | *95 % CI* | |  | *coef.* | *95 % CI* | |  | *coef.* | *95 % CI* | |  | *coef.* | *95 % CI* | |  |
| 0 Nights | Ref 1 |  |  |  | Ref 1 |  |  |  | Ref 1 |  |  |  | Ref 1 |  |  |  |
| 1-3 nights | 0.25 | 0.08 | 0.41 |  | 0.33 | 0.06 | 0.60 |  | -0.01 | -0.16 | 0.13 |  | -0.19 | -0.41 | 0.02 |  |
| > 3 nights | 0.12 | -0.22 | 0.46 |  | 0.02 | -0.55 | 0.58 |  | -0.30 | -0.60 | 0.00 |  | 0.09 | -0.36 | 0.54 |  |
| P-value* | 0.012 | | |  | 0.049 | | |  | 0.14 | | |  | 0.17 | | |  |
|  |  |  |  |  |  |  |  |  |  |  |  |  |  |  |  |  |
| **Table S2: Associations between nighttime smartphone use and mental health and well-being at follow-up** | | | | | | | | | |  |  |  |  |  |  |  |
|  | **Perceived stress** | | | | **Depressive symptoms** | | | | **Loneliness** | | | | **Satisfaction with life** | | | |
| Nights with smartphone use | *coef.* | *95 % CI* | | *P-value for interaction*** | *coef.* | *95 % CI* | | *P-value for interaction*** | *coef.* | *95 % CI* | | *P-value for interaction*** | *coef.* | *95 % CI* | | *P-value for interaction*** |
| 0 Nights | Ref 1 |  |  | 0.77 | Ref 1 |  |  | 0.22 | Ref 1 |  |  | 0.81 | Ref 1 |  |  | 0.92 |
| 1-3 nights | 0.08 | -0.14 | 0.30 |  | -0.06 | -0.37 | 0.25 |  | -0.02 | -0.19 | 0.15 |  | 0.19 | -0.04 | 0.42 |  |
| > 3 nights | -0.05 | -0.52 | 0.41 |  | -0.75 | -1.40 | -0.10 |  | -0.23 | -0.60 | 0.13 |  | 0.19 | -0.29 | 0.67 |  |
| P-value* | 0.74 | | |  | 0.08 | | |  | 0.45 | | |  | 0.24 | | |  |
| All scales are rescaled to 0-10 |  |  |  |  |  |  |  |  |  |  |  |  |  |  |  |  |
| All estimates are adjusted for age, gender, cohabitation, personality (extroversion, neuroticism), social network score | | | | | | | | | | |  |  |  |  |  |  |
| 95% CI: 95 % confidence intervals | |  |  |  |  |  |  |  |  |  |  |  |  |  |  |  |
| Ref: reference group |  |  |  |  |  |  |  |  |  |  |  |  |  |  |  |  |
| *P-value from F-test |  |  |  |  |  |  |  |  |  |  |  |  |  |  |  |  |
| **P-value for the interaction between nighttime smartphone use and follow-up time | | | | | | |  |  |  |  |  |  |  |  |  |  |

**Baseline associations where population is restricted to respondents at follow-up**


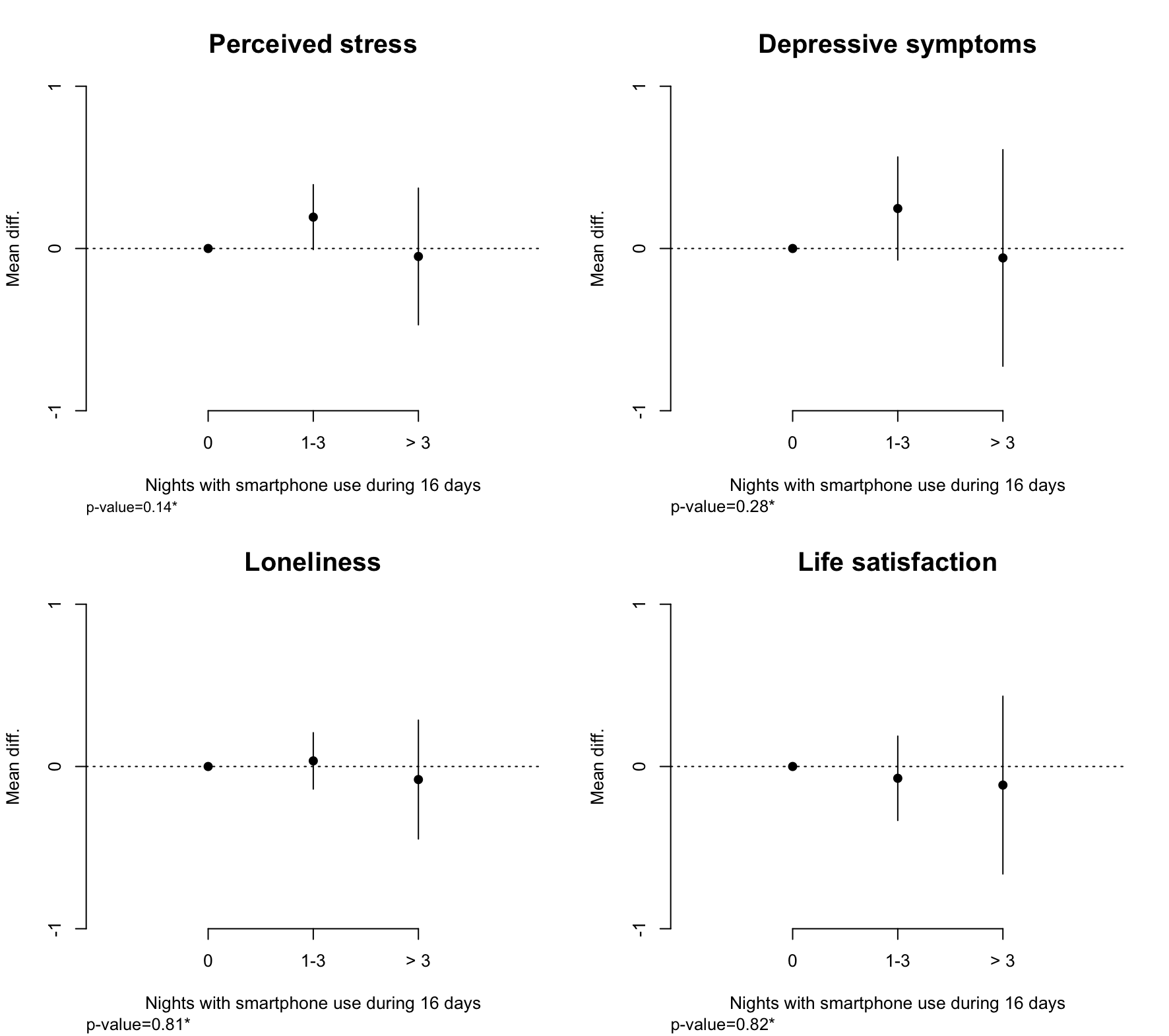


Figure S1: Associations between nighttime smartphone use and changes in mental health at baseline where population is restricted to population at follow-up

All estimates are adjusted for age, gender, personality (neuroticism and extroversion), co-habitation, social network score

*P-value from F-test
